# Influence of microbiota on the growth and gene expression of *Clostridioides difficile* in an in vitro coculture model

**DOI:** 10.1002/mbo3.70001

**Published:** 2024-10-15

**Authors:** Elisa Martinez, Noémie Berg, Cristina Rodriguez, Georges Daube, Bernard Taminiau

**Affiliations:** ^1^ Department of Food Sciences, Food Microbiology Fundamental and Applied Research for Animals & Health (FARAH), Faculty of Veterinary Medicine, University of Liege Liege Belgium; ^2^ Instituto de Investigación Biomédica de Málaga‐IBIMA Málaga Spain; ^3^ Unidadde Gestión Clínica de Aparato Digestivo Hospital Universitario Virgen de laVictoria Málaga Spain

**Keywords:** *Clostridioides difficile*, gastrointestinal microbiome, gene expression, growth, in vitro technique, metabolism

## Abstract

*Clostridioides difficile* is an anaerobic, spore‐forming, Gram‐positive pathogenic bacterium. This study aimed to analyze the effect of two samples of healthy fecal microbiota on *C. difficile* gene expression and growth using an in vitro coculture model. The inner compartment was cocultured with spores of the *C. difficile* polymerase chain reaction (PCR)‐ribotype 078, while the outer compartment contained fecal samples from donors to mimic the microbiota (FD1 and FD2). A fecal‐free plate served as a control (CT). RNA‐Seq and quantitative PCR confirmation were performed on the inner compartment sample. Similarities in gene expression were observed in the presence of the microbiota. After 12 h, the expression of genes associated with germination, sporulation, toxin production, and growth was downregulated in the presence of the microbiota. At 24 h, in an iron‐deficient environment, *C. difficile* activated several genes to counteract iron deficiency. The expression of genes associated with germination and sporulation was upregulated at 24 h compared with 12 h in the presence of microbiota from donor 1 (FD1). This study confirmed previous findings that *C. difficile* can use ethanolamine as a primary nutrient source. To further investigate this interaction, future studies will use a simplified coculture model with an artificial bacterial consortium instead of fecal samples.

## Graphical abstract

The presence of microbiota did not affect the growth of *Clostridioides difficile* in this study. However, it did influence the expression of *C. difficile* genes related to sporulation, germination, and virulence, which are crucial for the transmission of the pathogen. In the presence of microbiota, *C. difficile* activates defence mechanisms to survive competition, such as adapting to an iron‐limited environment and utilizing ethanolamine metabolism.

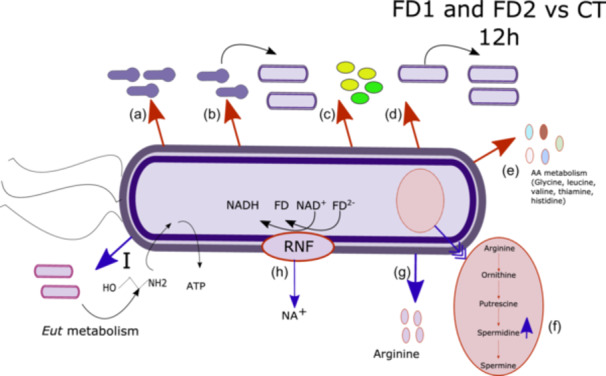

## 1 INTRODUCTION

*Clostridioides difficile* is an anaerobic spore‐forming Gram‐positive bacterium. The colonization of *C. difficile* in the gut can lead to pseudomembranous colitis or transient asymptomatic carriage. This bacterium can produce three different toxins (TcdA, TcdB, and CDT). In Belgium, 2294 cases of CDI were reported in 2022 (Callies et al., 2024). In 2022, the five most frequent polymerase chain reaction (PCR)‐ribotypes in Belgium were 014 (13%), 002 (10.0%), 078 (8.2%), 102 (8.2%), and 020 (6.4%) (Callies et al., 2024).

Many in vitro models for studying *C. difficile* growth have been described (Best et al., 2012; Chilton et al., 2014; Ewin et al., 2023; Freeman et al., 2007; Shaban et al., 2018). Two different in vitro models exist: bacterial fermentation models and bacterial–human interaction models (Ewin et al., 2023). Nevertheless, there is currently no established transwell coculture model specifically designed to investigate the impact of the gut microbiota on *C. difficile* growth. A simplified coculture model has been described for *Salmonella* Typhimurium growth (Avendaño‐Pérez et al., 2015).

New technologies for analyzing *C. difficile* gene expression and understanding the behavior of this bacterium have emerged. In recent years, transcriptome analysis has revealed genes essential for the physiology and pathology of *C. difficile* (Dembek et al., 2015; Neumann‐Schaal et al., 2019). The effects of the exponential and stationary phases of *C. difficile* growth on gene expression have been studied in vitro (Hofmann et al., 2018). In the stationary phase, the expression of certain genes associated with motility and iron transport was downregulated, while the expression of other genes associated with the Stickland reaction, the fermentation reaction, and the serine pathway was upregulated. The oxidative pathway of Stickland metabolism is used in the late stationary phase (Hofmann et al., 2018). *C. difficile* 630△*erm* in a nongrowing state had reduced lipid metabolism, spermine biosynthesis, glycolysis, riboflavin biosynthesis and CoA biosynthesis in the stationary phase (Hofmann et al., 2018). In the presence of oxygen, *C. difficile* showed an increase in cysteine metabolism and the de novo synthesis of cysteine (Neumann‐Schaal et al., 2018). Another study showed a relationship between proline fermentation and *C. difficile* growth (Lopez et al., 2019). Low zinc availability increased the expression of genes encoding proline fermentation products (Lopez et al., 2019).

To understand the behavior of *C. difficile*, knowledge of its metabolic pathways is essential (Neumann‐Schaal et al., 2019). The fermentation of amino acids (leucine, valine, isoleucine, phenylalanine, etc.) in *C. difficile* occurs via the Stickland metabolism pathway. The reduction of proline and glycine or the degradation of arginine to ornithine occurs via a modified Stickland pathway. Glutamate and arginine are converted to ornithine via the carbamoyl transferase and acetylornithine aminotransferase pathways (Johnstone & Self, 2022; Pruss et al., 2022). The first step involves the transamination of an amino acid to its corresponding 2‐oxo acid. The second step is either an oxidative or reductive pathway. The final step is the cleavage of the CoA thioester with ATP formation (Neumann‐Schaal et al., 2019). *C. difficile* produces acetate, lactate, propionate and butyrate through pyruvate and acetyl‐CoA via the reductive Stickland pathway (Neumann‐Schaal et al., 2019).

In this study, an in vitro coculture model was developed using a transwell plate: *C. difficile* spores and nutrient media were in the inner compartment, and nutrient media with microbiota from fecal donors were in the outer compartment. The first objective of this work was to analyze the effect of the presence of microbiota on *C. difficile* gene expression. The second objective was to compare the *C. difficile* gene expression at two time points (12 h and 24 h) in the presence of microbiota.

## 2 MATERIAL AND METHODS

### 2.1 Bacterial strain and spore production

The *C. difficile* strain used in this study belonged to PCR‐ribotype 078 and was selected from the bacterial collection available in our laboratory. The strain was isolated from piglet feces (Rodriguez et al., 2012). The strain was laboratory‐specific strain‐labeled (S0756) and stored frozen (−80°C) in a solution of 20% glycerol in 80% BHI (VWR 24388.295; Brain Heart Infusion; Oxoid CM1032).

For spore production and purification, *C. difficile* spores were prepared according to the following protocol. Briefly, the strain stored at ‐80°C was placed in antibiotic‐free BHI broth and incubated at 37°C for 7 days under strict anaerobic conditions. After incubation, the bacteria were harvested by sample centrifugation (Eppendorf; Centrifuge 5810 R) (20 min at 3900 rpm) and the spores were selected via ethanol treatment (1 mL; 95% ethanol at room temperature) and incubated for 1 h. Next, two washing steps with a sterile 0.9% saline solution were performed (5 min at 3900 rpm), and the final suspension was stored in 1 mL of a sterile 0.9% saline solution at 4°C. To determine the final spore concentration, 10‐fold serial dilutions of a sterile 0.9% saline solution were made, and 100 µL of each dilution was spread on blood agar (Thermo Fisher Scientific; PB5039A) and on homemade cycloserine cefoxitin fructose agar taurocholate (CCFAT) (Delmée et al., 1987). The plates were incubated anaerobically for 48 h at 37°C before enumeration.

### 2.2 Genome sequencing and analysis

A 10‐μL aliquot of the stored bacteria was grown in BHI broth for genome sequencing. The solution was incubated anaerobically at 37°C for 48 h and further sub‐cultured on blood agar. The DNA extracted from a colony was sequenced by two methods: Illumina MiSeq (GIGA, sequencing center) (Martinez et al., 2022) and Oxford Nanopore Sequencing with Ligation Sequencing Kit “flow cell R9.4.1” (GIGA, sequencing center). The first analysis was performed on the Illumina MiSeq raw reads and published in Martinez et al. (2022). The raw reads from Oxford nanopore sequencing were used, and de novo assembly was performed using Trycycler software (v0.5.3) (Wick et al., 2021). Trycycler generated four subgroups and made four assemblies with Flye (v2.9.1) (Kolmogorov et al., 2019), Miniasm/Minipolish (v0.3/v0.1.3) (Li, 2016; Wick & Holt, 2019) and Raven (v1.7.0) software (Vaser & Šikić, 2021). Clustering and consensus were performed using Trycycler software with the Mash distance (Ondov et al., 2016). The final step of Polypolish was performed using raw reads from Illumina MiSeq to improve the assembly quality. Annotation was performed using the NCBI—Prokaryotic Genome Annotation Pipeline (Tatusova et al., 2016) and the circularized genome assembly is available in GenBank in the Bioproject PRJNA716140. A fully annotated genome analysis was performed in BV‐BRC (version 3.28.21) (Olson et al., 2023).

### 2.3 Fecal collection and dilution

Fecal samples were collected from two healthy male donors aged 66 and 26 years (called FD1 and FD2, respectively), who had no history of antibiotic therapy in the 2 months before recruitment.

The absence of *C. difficile* was determined by both direct culture and fecal enrichment culture as described previously (Rodriguez et al., 2012). A *C. difficile* test kit (Thermo Scientific and Oxoid; DR1107A) was used to examine the colonies from the fecal samples. Fecal samples containing morphologically suspected *C. difficile* colonies were discarded. Fresh feces were diluted with sterile phosphate buffer and stomached for 120 s at room temperature (Interscience; Bagmixer 400) to obtain a final solution of 20% filtered feces. Then, glycerol (20% of the final solution) was added, and the fecal samples were kept at −80°C until further testing.

Amplicon sequencing of these fecal samples was performed in a previous study (Martinez et al., 2022). These patients were referred to as donors 4 and 3 in a previous study. For this study, they were selected based on the age of the donor and the result of the ecological analysis (Martinez et al., 2022). Donor 4 was over 65 years old and Donor 3 was 26 years old. Donor 4 feces were mainly composed of *Faecalibacterium*, *Lachnospiraceae*_ge, and *Blautia*. Donor 3 feces were mainly composed of *Prevotella*_9, *Faecalibacterium*, *Lachnospiraceae*_ge, and *Subdoligranulum*. For this study, feces from Donors 4 and 3 were referred to as feces donor 1 (FD1) and feces donor 2 (FD2), respectively.

### 2.4 Bacterial coculture in an anaerobic chamber

Three experiments were performed: one experiment was performed without microbiota content, called “CT”; one experiment included microbiota from Donor 1 (age: 66 years), named “FD1”; and one experiment included microbiota from Donor 2 (Age: 26 years), named “FD2” (Martinez et al., 2022). Two solutions were used: solution A was used for the inner compartment called “IN”, and solution B was used for the outer compartment called “OUT”. These two solutions were prepared with 30 mL of nutritional medium. This medium contained 70% feed (ProDigest, commercial Adult L‐SHIME growth medium with starch, PDNM001B) and 30% pancreatic juice (PJ) (Prodigest, pancreatic enzymes, PDDE001; bile salts, PDDE002; NaHCO_3_, PDDE007). The PJ tubes were filtered through a 0.22 µm filter and placed in an anaerobic jar at 4°C for 12 h. The Feed tubes were heated to 95°C to degas. In the control (CT) experiment, a concentration of 10^3^ UFC mL^−1^ of *C. difficile* spores was added to solution A. In Donor 1 (FD1) and Donor 2 (FD2), a concentration of 10^3^ UFC mL^−1^ of *C. difficile* spores was added to Solution A and 1 mL of diluted feces from Donors 1 and 2 was added to Solution B. A culture transwell plate was used with a 0.4 µm membrane (Greiner, Thincert® cell culture insert for six wells, 657641). Four milliliters of Solution B were added to the outer compartment (OUT) and then, 2 mL of Solution A were subsequently placed in the inner compartment (IN). Three biological replicates of each condition were collected at 1, 6, 12, and 24 h. These experiments were repeated twice. The control (CT) experiment was the control. The microbiota were present in FD1 and FD2.

### 2.5 DNA extraction, RNA extraction, and cDNA conversion

Total DNA extractions were performed using QiAamp Power Fecal Pro DNA (Qiagen). Total RNA was extracted using RNeasy PowerMicrobiome (Qiagen). Two DNase treatments were performed for all the samples, and a control PCR targeting the 16S rRNA gene was performed after extraction to verify the absence of DNA. The length of the fragment was 1500 bp, and the primers used are listed in Appendix: Table A4 (Minutillo et al., 2023). All extractions were performed following the manufacturer's instructions. Then, the cDNA of each RNA extract was synthesized using the QuantiTect Reverse Transcription Kit (Qiagen). The RNA concentration was normalized to 50 ng before the manipulation. Fifty‐ng of RNA extract, 2 µL of gDNA wipeout buffer and molecular water were added to achieve a final volume of 14 µL. The samples were heated at 42°C for 2 min. Then, 1 µL of Quantiscript Reverse Transcriptase, 4 µL of Quantiscript RT Buffer and 1 µL of RT primer mixture were added to each sample, which was heated at 42°C for 30 min and at 95°C for 3 min. cDNA was stored at −20°C.

### 2.6 *C. difficile* enumeration

Two methods were used to monitor the growth of *C. difficile*: microbiological analysis and genetic analysis. Microbiological analysis was performed on the CCFAT in the second experiment (Delmée et al., 1987). A specific qPCR targeting the 16S rRNA gene of *C. difficile* (157 bp, F64‐R220) described in Mutters et al. (2009) was performed for all the inner compartment (IN) samples. Standard DNA was obtained from the purified PCR products and quantified using PicoGreen (Thermo Fisher Scientific). qPCR was performed using CFX96 (Bio‐rad) in a final reaction volume of 12.5 µL. The mixture contained 1x master mix Takyon Rox Probe 2x MasterMix (Eurogentec), 300 nM primers, 250 nM probe, 3.19 µL of molecular water and 2 µL of DNA. Genome equivalent values were deduced from CT to 16S copy‐transformed values considering 12x 16S rRNA gene copies per genome and converted to bacterial genome concentrations. These results are presented as “log_10_ (bacterial genome mL^−1^)” in Section 3. One‐way analysis of variance (ANOVA) was performed with Prism software v9.1.1 to compare the three conditions. Growth potential was calculated from microbiological analysis and the growth monitoring was performed using genetic data due to its best repeatability in this study.

### 2.7 *C. difficile* transcriptome analysis

Two experiments were performed for each of the three conditions (CT, FD1, and FD2) in three biological replicates at both 12 h and 24 h. The RNA extracts from the inner compartment (IN) were transferred on ice to a ULiège‐GIGA platform. A QC analysis (Agilent, 2100 bioanalyzer) was performed for all the samples. QC identified the samples for which the RNA quantity was sufficient to perform RNA‐Seq. The RNA integrity number (RIN) assessments were conducted on all the samples. The RIN is a tool used to estimate the integrity of total RNA samples (Mueller et al., 2016). A minimum of 90 pg/µL was necessary to perform the RNAseq using Illumina Stranded Total RNA Ligation with Ribo‐zero Plus (Illumina; Novaseq S4 V1.5 300 cycles). One to ten nanograms were subjected to RNAseq sequencing. The quantity of total RNA was sufficient to perform RNA sequencing. The raw reads (*n* = 27) are available in GenBank in the Bioproject (accession number PRJNA1023484). Appendix: Table A1 provides a correspondence table between the library ID in GenBank and the names used in this study.

The fastq files were obtained after RNAseq. The first step was to merge files R1 and R2 using Mothur (version 1.39.5). The second step was to evaluate the quality of trimming and adapter removal using FASTp (version 0.20.1). The third step involved removing ribosomal RNA using SORTMERNA (version 2.1b). The next step was to map the reads on our reference genome using subread (version 2.0.1) and subjunc (version 2.0.1) tools. The last step involved counting the reads using FeatureCount (v2.0.1). Using the count table, DESeq2 (v1.36.0) analysis was realized in R studio (v4.2.2). First, DESeq2 applied standard normalization to the data (median of ratios method). Second, DESeq2 was used to calculate the log_2_fold change of each gene between two conditions (FD1 vs. CT and FD2 vs. CT). Then, all the tables were integrated into a Perl program to enrich the data and obtain the KO numbers and modules.

The Kyoto Encyclopedia of Genes and Genomes (KEGG) is an encyclopedia of genes and genomes that defines a gene by its metabolic function in a pathway. The KEGG database assigns a KO (KEGG orthology) number to a set of genes with a similar metabolic/chemical function.

A module was defined as a gene set with several KO numbers and the same functional unit. Using GhostKOALA, a database‐internal annotation system, KEGG was used to assign KO numbers to protein sequences in FASTA (Kanehisa, 2000; Kanehisa, Sato, Kawashima, et al., 2016; Kanehisa, Sato et al., 2016). These KOs are associated with one or more modules.

Two different analyzes were performed to analyze the data. First, the effect of the presence of microbiota on *C. difficile* gene expression was examined (Analysis 1) on the six 12 h replicates of the two experiments. Similar patterns of gene expression in the two fecal samples were analyzed together in Analysis 1, and gene expression in the two fecal samples was compared with that of the control (FD1 vs. CT and FD2 vs. CT). Secondly, in the second experiment, the time effect on *C. difficile* gene expression (12 h vs. 24 h) was investigated on the three replicates of 12 h and 24 h (Analysis 2) (see Figure 2).

From the FeatureCount outputs, normalized counts were calculated using DESeq2. Several complex heatmaps were created for the expression of all the genes in the two analyzes using R studio v4.2.2, as shown in Figure 3. With DESeq2 outputs, several volcano plots were realized using R studio (CT 24 h vs. 12 h, FD1 24 h vs. 12 h, FD2 24 h vs. 12 h; FD1 vs. CT 12 h and 24 h, FD2 vs. CT 12 h and 24 h) in Figure 4.

### 2.8 Study of *C. difficile* virulence and metabolism

Specific qPCR targeting genes of *C. difficile* via cDNA were performed for the inner compartment (IN) samples. Details about primers are provided in Appendix: Table C1. Standard DNA was obtained from the purified PCR products and quantified using PicoGreen (Thermo Fisher Scientific). The purified PCR products were subsequently sequenced to verify the amplicon. Ten‐fold dilutions of these standard DNAs were performed to generate a standard curve. qPCR was performed using CFX96 (Biorad) in a final reaction volume of 12.5 µL. The mixture for the multiplex (*gluD*, *tcdA*, and *tcdB)* contained 1x master mix Takyon Rox Probe 2x MasterMix (Eurogentec), 300 nM primers, 200 nM probes, 3.19 µL of molecular water and 2 µL of DNA. Following 2 min of activation at 50°C and 5 min at 95°C, the reactions were amplified in 40 cycles of 15 s at 95°C and 45 s at 57°C. The second mixture (*gluD*, *rnfG*, *eutA*, *eutB*, and *flaA*) contained 1x master mix Takyon Rox Syber 2x MasterMix (Eurogentec), 300 nM primers, 50 ng of bovine serum albumin (BSA), 1.25 µL of molecular water and 2 µL of DNA. Following 2 min of activation at 50°C and 5 min at 95°C, the reactions were amplified in 40 cycles of 15 s at 95°C and 45 s at variable temperatures.

To validate RNA‐Seq data, two analyzes were performed on data from qPCR. The first analysis consisted of 2^−(△△CT)^, in which the genes of interest were *tcdA*, *tdcB*, *flaA*, *rnfG*, *eutA*, and *eutB* and the housekeeping gene was *gluD* (Livak & Schmittgen, 2001). The Donor 1 (FD1) and Donor 2 (FD2) samples are the focus of interest. The second element of the conditions is the control condition (CT or FD2 samples). The second method involved log_2_ (genes of interest copies mL^−1^) normalization to the expression of *gluD*. To standardize the comparison in the graph, a log_2_ transformation was applied to all the samples.

## 3 RESULTS

### 3.1 *C. difficile* genome characteristics

**Figure B1:**
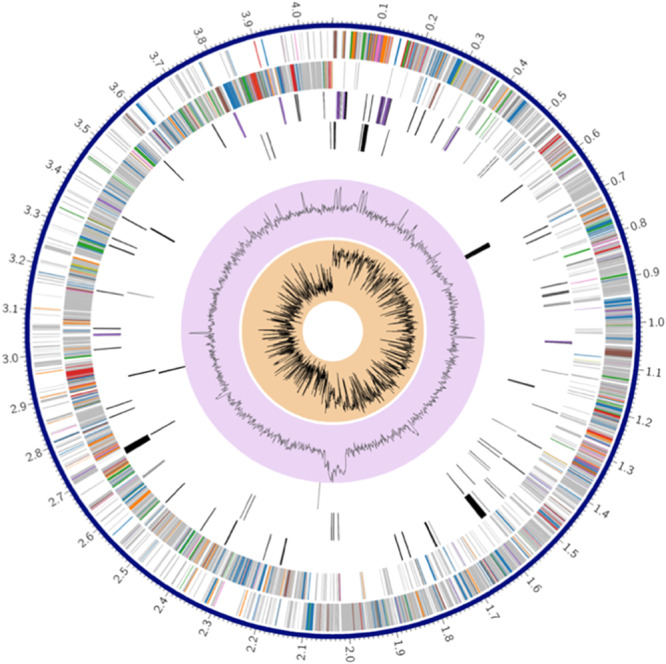
Circularization of the S0756_078 *C. difficile* genome This figure was generated using Trycyler (Wick et al., 2021) (software v0.5.3) and illustrated by BV‐BRC platform (Olson et al., 2023) (v3.28.9). The different layers from the outside to the inside. Layer 1 represents the CDS forward; Layer 2 represents the CDS reverse; Layer 3 is the RNA genes, the Layer 4 is the antimicrobial resistance (AMR) genes, the Layer 5 is the virulence factors (VF) genes, Layer 6 is the GC content and Layer 7 is the GC skew. The complete genome comprises 4,071,782 bp which contains 3740 genes, including 3541 CDCs. (with protein), 35 rRNAs and 90 tRNAs. This strain contains both virulence operons (PaLoc and CdtLoc). The ribotype 078 is commonly found in animals (Rodriguez et al., 2012) and is the third most common in European hospitals (Abdrabou et al., 2022). The GC content was 29.05%. Ninety‐ nine genes were involved in the stress response, defence, and virulence. This strain has 12 copies of the 16S rRNA gene.

**Figure B2:**
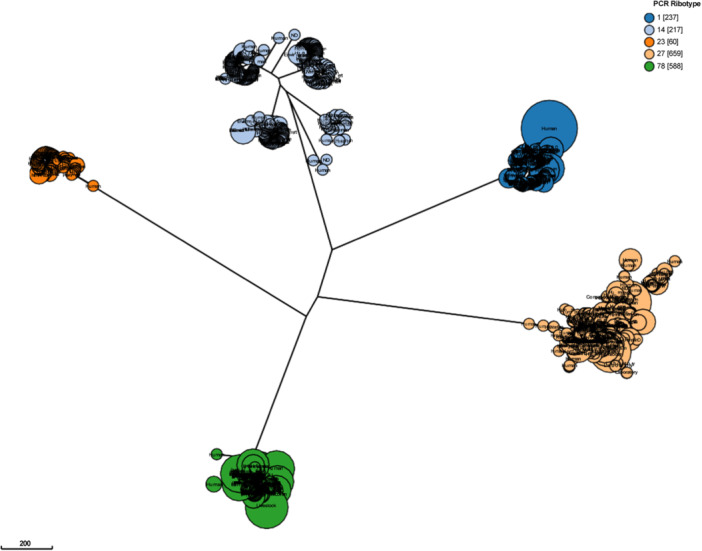
Grape tree of the five most common PCR‐ribotypes of *Clostridioides difficile*. Phylogenetic grape tree generated using the 2556 loci cgMLST+hierCC V1 design in Enterobase. The analysis was performed with the five most common PCR‐ribotype 001 (*n* = 237), 014 (*n* = 217), 023 (*n* = 60), 027 (*n* = 659), and 078 (*n* = 588) (Frentrup et al., 2020; Zhou et al., 2018). Our strain (S0756) is located in the PCR‐ribotype 078 group.

One strain of *C. difficile* PCR‐ribotype 078 was isolated (Rodriguez et al., 2012) and sequenced. The *C. difficile* genome was circularized using Trycyler (Wick et al., 2021) (software v0.5.3). Appendix B: Figure B1 shows a representation of *C. difficile* circularization illustrated by the BV‐BRC platform (Olson et al., 2023) (v3.28.9). This PCR‐ribotype contained both virulence operons (PaLoc and CdtLoc). The genome annotation is available in the GenBank repository under the PRJNA716140 bioproject and GCA_017 592 625.2 assembly. A grape tree of the five most common PCR‐ribotypes found in Europe, including the strain used in this study, is shown in Appendix B: Figure B2.

### 3.2 *C. difficile* enumeration and coculture bacterial model

**Figure 1:**
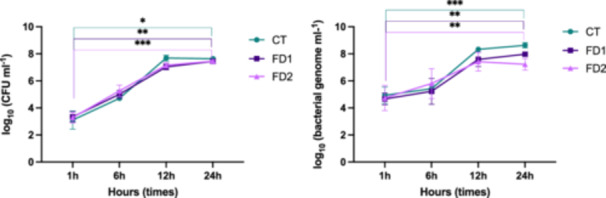
Growth curve of *Clostridioides difficile* in transwell plate in three conditions (control (CT), feces donor 1 (FD1), feces donor 2 (FD2)) at different time points (1, 6, 12, and 24 h). The results of qPCR targeting 16S rRNA gene specific for *C. difficile* are expressed as log_10_ (bacterial genome mL^−1^), and on the left, the results of classical microbiology are expressed as log_10_ (CFU mL^−1^). (A) The results of the blood agar count of *C. difficile* are represented for the three conditions in one graph (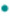) CT (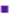) FD1 (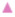) FD2. (B) The results of 16S *C. difficile* counts are presented for the three conditions in one graph (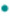) CT (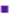) FD1 (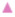) FD2. *Source:* These growth curves were generated using Prism software (v9.1.1).

Cultures were performed using a transwell plate. *C. difficile* PCR‐ribotype 078 spores and medium were introduced into the inner compartment (IN). In the outer compartment (OUT), medium and feces samples were added under the following conditions: feces from Donor 1 (FD1) and feces from Donor 2 (FD2). Under the control condition (CT), no feces were added, and only the medium was added to the outer compartment (out). The results of the microbiological counts and the specific qPCR targeting the 16S rRNA gene of *C. difficile* are similar. First, there was an exponential phase observed up to 12 h, after which the growth curves reached a plateau (Figure 1). The initial concentrations of *C. difficile* in the transwell “in” were 3.1 (±0.66) log_10_ (CFU mL^−1^), 3.3 (±0.40) log_10_ (CFU mL^−1^) and 3.3 (±0.40) log_10_ (CFU mL^−1^) for control (CT), Donor 1 (FD1) and Donor 2 (FD2), respectively. No significant difference among the three conditions was detected using one‐way ANOVA in Prism 9. Among the three conditions, the difference between 1 h and 24 h time points was significant. The concentrations of *C. difficile* at 24 h in the transwell “in” were 7.6 (±0.06) log_10_ (CFU mL^−1^), 7.4 (±0.14) log_10_ (CFU mL^−1^) and 7.5 (±0.09) log_10_ (CFU mL^−1^) for control (CT), Donor 1 (FD1), and Donor 2 (FD2), respectively (Figure 1).

### 3.3 *C. difficile* transcriptomic analysis

**Figure 2:**
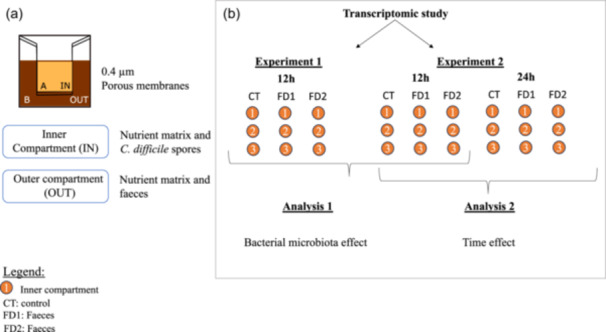
Schematic representation of experimental design. (a) Composition of the transwell plate with inner compartment (IN) (*Clostridioides difficile* spores, feed, and PJ (nutrient matrix)) and outer compartment (OUT) (feces, feed, and PJ (nutrient matrix)) separated by a membrane with 0.4 µm pores. (b) Two experiments were performed according to the same protocol and were designated as Experiments 1 and 2. There are three conditions: CT (control), FD1 (feces from Donor 1) and FD2 (feces from Donor 2). Samples from the inner compartment were sequenced at 12 h for Experiment 1 and at 12 h and 24 h for Experiment 2. Two bioinformatic analyzes were performed on the transcriptomic data to investigate the effects of bacterial microbiota and incubation time. The first analysis (1) was performed on the six replicates at 12 h and the second analysis (2) on three replicates at 12 h versus 24 h. Data from FD1 and FD2 were compared with the control in Analysis 1 and data between 12 h and 24 h from the same condition were compared in Analysis 2.

RNA sequencing was performed on samples from the inner compartment (IN). Details of the RNA integrity number (RIN), quality control (QC) value (pg/µL), mapping and genome coverage are provided in Appendix A: Table A2. Two experiments were performed for each of the three conditions (CT, FD1, and FD2) at both 12 h and 24 h. In the first experiment, bacterial contamination was detected in the inner compartment of the 24 h samples. These samples were excluded from the further analysis. Two RNAseq analyzes were performed. First, the impact of the presence of microbiota on gene expression of *C. difficile* was investigated. Analysis 1 was performed on the six biological replicates of 12 h of both analyzes. For the data analysis, gene expression in the two fecal samples was compared to that of the control (FD1 vs. CT and FD2 vs. CT). Similar patterns of gene expression in the two fecal samples were analyzed together. The pattern of differential gene expression between the two fecal samples was also analyzed. Second, the impact of time on gene expression of *C. difficile* was studied (12 h vs. 24 h). Analysis 2 was performed on the 12 h and 24 h samples of the second experiment. Figure 2 shows the details of the experimental design. All reads are available in the GenBank repository under PRJNA103484. All the nonsignificant and significant data (log_2_fold change) are available in Supporting Information: Tables S1 and S2: https://zenodo.org/doi/10.5281/zenodo.13121217.

#### 3.3.1 Global gene expression patterns

**Figure 3:**
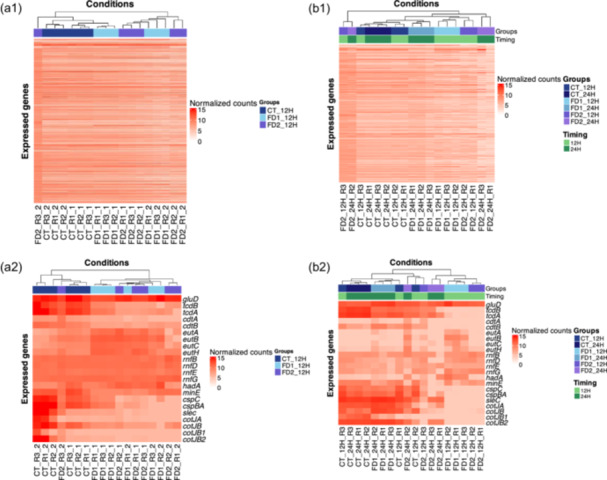
Heatmap of normalized counts of whole *Clostridioides difficile* S0756 genes using complexHeatmap in R studio v4.2.2. (a1) Heatmap representing the expression of the three conditions (CT, FD1, and FD2) at time 12 h of Analysis 1 (*n* = 6). (a2) Heatmap showing the expression of the genes of interest and their normalized values. The experimental conditions were clustered according to the similarity of their expressions. (b1) Heatmap showing the expression of the three conditions (CT, FD1, and FD2) at 12 h and 24 h of Analysis 2 (*n* = 3). Two headbands are shown: the first one represents the groups and the second one represents the time. (b2) Heatmap representing the expression of the genes of interest and their normalized counts. The two headbands represented in b1 and b2 follow the same organization: the first one represents the group factor and the second one represents the time factor.

The global gene expression patterns under different conditions were clustered as a function of the conditions (CT vs. microbiota) (Figure 3a1). In the absence of microbiota, global gene expression was constant and repeatable between the two analyzes. When a microbiota was present, the expression was variable but homogeneous within the same analysis. The global gene expression across various conditions and different time points revealed that control (CT) and Donor 1 (FD1) exhibited homogeneous expression patterns over time (Figure 3b1). The Donor 2 (FD2) analysis showed heterogeneous expression.

Specific genes were selected based on their involvement in *C. difficile* metabolism and virulence (*gluD*, *tcdB*, *tcdA*, *cdtA*, *cdtB*, *eutA*, *eutB*, *eutC*, *eutH*, *rnfB*, *rnfD*, *rnfE*, *rnfG*, *hadA*, *minE*, *cspC*, *cspBA*, *scleC*, *cotJA*, *cotJB*, *cotJB1*, and *cotJB2*). The expression of specific genes of interest clustered in different conditions (CT vs. microbiota) (Figure 3a2). The expression of specific genes of interest across various conditions and different times clustered at different time points (12 h vs. 24 h) and different conditions (CT vs. microbiota) (Figure 3b2).

**Figure 4:**
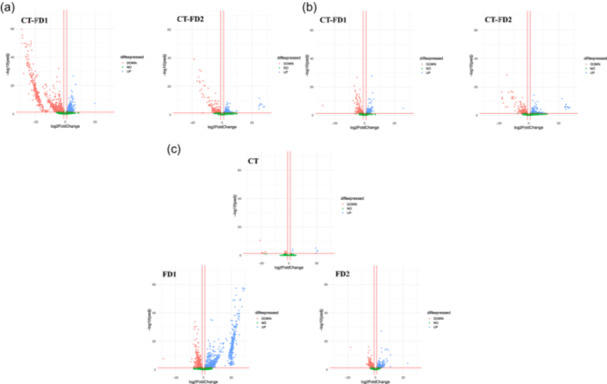
Effect of the presence of bacterial microbiota on *Clostridioides difficile* gene (a and b) and effect of the time on *C. difficile* gene (c). (a) Bacterial microbiota effects at 12 h using Experiment 2. The DESeq2 analysis has compared the normalized data between FD1 and FD2 at 12 h (*n* = 3), with CT as baseline. This is a representation of the significant genes that are downregulated or upregulated in FD1/FD2 compared to no bacterial microbiota. Most of the FD1/FD2 genes are downregulated compared to the control. (b) Bacterial microbiota effect at 24 h using Experiment 2. The DESeq2 analysis has compared the normalized data between FD1/FD2 and CT at 24 h (*n* = 3), with CT as baseline. This is a representation of the significant genes that are downregulated or upregulated in FD1/FD2 compared to no bacterial microbiota. (c) Time effect using Analysis 2. The DESeq2 analysis has compared the normalized data between 12 h and 24 h in the same condition, with 12 h as the baseline for this analysis. This is a representation of the significant genes that are downregulated or upregulated between the two times. Most of the FD1 genes are upregulated in the presence of bacterial microbiota. Volcano plots created using R studio (v4.2.2). The x‐axis shows the log_2_fold change. The y‐axis shows the −log_10_ (*p* adj). The more significant the gene is, the higher the point. Three red lines are shown in the graph, the x‐axis is the log_2_fold change threshold (−1 and 1) and the y‐axis is the significance level (*p*‐value < 0.05). (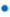) UP: genes upregulated (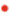) DOWN: genes downregulated (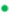) NO: nonsignificant genes.

**Figure A1:**
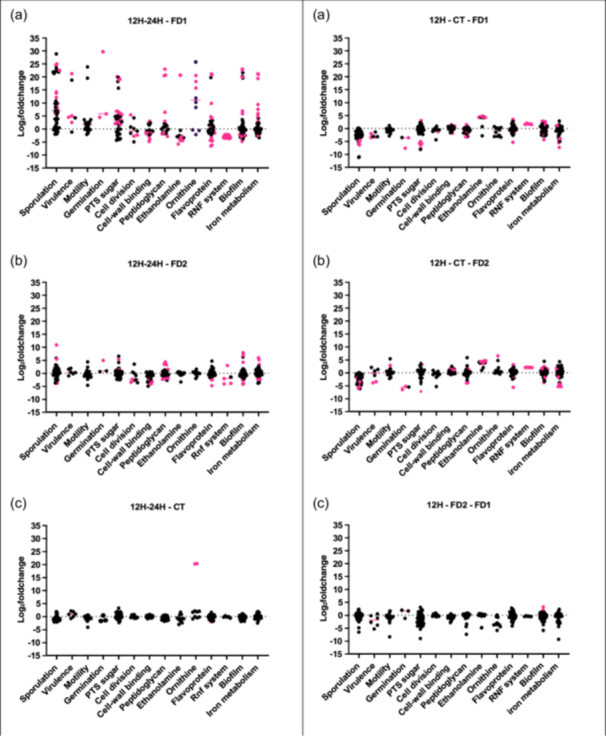
Main genes of categories shown in log_2_ fold change. (1) Log_2_ fold change of several genes grouped into categories between 12h and 24h in the three conditions of Analysis 2; (a) FD1 condition; (b) FD2 condition; (c) no feces condition. Each gene is represented by a dot (●). The pink dots are significant genes. The complete lists of genes are provided in Supporting Information: Table S3. (2) Log_2_ fold change of several genes grouped into categories at 12h (*n* = 6) from Analysis 1; (a) FD1 versus CT condition; (b) FD2 versus CT condition; (c) FD1 versus FD2 conditions. Each gene is represented by a dot (●). The pink dots are significant genes. The full lists of genes are provided in Supporting Information: Table S3.

Significant differences in gene transcription between different conditions were determined using Deseq2 analysis from analysis 2 (12 h vs. 24 h, CT vs. FD1 at 12 h, CT vs. FD2 at 12 h, CT vs. FD1 at 24 h, CT vs. FD2 at 24 h) (Figure 4). Fourteen categories were chosen to understand the metabolism of *C. difficile* and their significance in these assays: sporulation, virulence, motility, germination, PTS sugar, cell division, cell wall binding, peptidoglycan synthesis, ethanolamine synthesis, ornithine synthesis, flavoprotein metabolism, the Rnf system, biofilm‐associated genes, and iron metabolism. Table 1 and Appendix A: Figure A1 show the variation in gene expression (log_2_fold change) between the different conditions. All log_2_fold changes and nonsignificant genes are listed in Supporting Information: Table S3: https://zenodo.org/doi/10.5281/zenodo.13121217.

**Table 1:** Genes expression (log_2_ fold change) variations between the different conditions tested.

Metabolism		Proteins name	Gene ID	Conditions								
				FD1 vs. CT		FD2 vs. CT		FD1 vs. FD2		24 h vs. 12 h		
				12 h	24 h	12 h	24 h	12 h	24 h	CT	FD2	FD1
Sporulation		Spore coat‐associated protein CotJA	J5O10_12400	*		*	*		*		*	*
		Spore coat‐associated protein CotJB	J5O10_07385	*		*					*	*
		RNA polymerase sporulation sigma factor SigE	J5O10_13765									*
		RNA polymerase sporulation sigma factor SigG	J5O10_13760	*		*	*		*			
		RNA polymerase sporulation sigma factor SigH	J5O10_00595				*		*			
		Sporulation transcription factor (*spo0A*)	J5O10_05835	*							*	
		Spore coat protein	J5O10_01620	*		*	*					*
		Stage II sporulation protein R	J5O10_18060				*					*
		Stage III sporulation protein AA	J5O10_05725			*						*
		Stage III sporulation protein AB	J5O10_05730									*
		Stage III sporulation protein AC	J5O10_05735			*						*
		Stage III sporulation protein AD	J5O10_05740									*
		Stage III sporulation protein AE	J5O10_05745									*
		Stage III sporulation protein AF	J5O10_05750									*
		Stage III sporulation protein AG	J5O10_05755			*	*		*			*
		Stage III sporulation protein AH	J5O10_05760	*		*	*		*			*
		Stage III sporulation protein D	J5O10_01190	*		*						*
		Stage IV sporulation protein A	J5O10_13680	*		*						*
		Stage V sporulation protein AC	J5O10_04050									*
		Stage V sporulation protein AD	J5O10_04055	*								*
		Stage V sporulation protein AE	J5O10_04060	*								*
		Stage V sporulation protein D	J5O10_13830	*							*	*
		Stage V sporulation protein E	J5O10_13810								*	
		Stage V sporulation protein T	J5O10_17750	*		*						*
		Sporulation peptidase YabG	J5O10_18085	*		*	*			*	*	*
		Sporulation protein YqfD	J5O10_12630			*						*
		Sporulation integral membrane protein YlbJ	J5O10_05600									*
		Sporulation integral membrane protein YtvI	J5O10_05075									*
		Sporulation protein YunB	J5O10_04095									*
Cell division		Cell division topological specificity factor	J5O10_05515	*	*		*				*	*
		Rod‐shape‐determining protein RodA	J5O10_05520		*				*		*	*
		Cell division protein sepF	J5O10_13645								*	*
		Cell division protein Ftsz	J5O10_13780								*	*
		UDP‐N‐acetylmuramoyl‐l‐alanyl‐d‐glutamate‐‐2%2 C 6‐diaminopimelate ligase *(murE)*	J5O10_13870									*
Cell wall‐binding		Cell wall‐binding protein Cwp2	J5O10_14580				*					*
		Cell wall‐binding protein Cwp5	J5O10_14555									*
		Cell wall‐binding protein Cwp7	J5O10_14535	*		*						
		Cell wall‐binding protein Cwp9	J5O10_14605	*	*							
		Cell wall‐binding protein Cwp13	J5O10_08755								*	
		Cell wall‐binding protein Cwp14	J5O10_14305						*			
		Cell wall‐binding protein Cwp18	J5O10_05085			*					*	
		Cell wall‐binding protein Cwp19	J5O10_14460								*	*
		Cell wall‐binding protein Cwp20	J5O10_07145								*	
		Cell wall‐binding protein Cwp22	J5O10_14150	*	*	*					*	*
		Cell wall‐binding protein Cwp25	J5O10_04395				*		*		*	
		Cell wall‐binding protein Cwp28	J5O10_10260									*
		Cell wall‐binding protein Cwp84	J5O10_14560		*						*	*
Peptidoglycan		Bifunctional protein GlmU	J5O10_17830				*		*		*	
		Phosphoglucosamine mutase	J5O10_01155		*		*				*	*
		Glutamine‐fructose‐6‐phosphate transaminase	J5O10_01160								*	
		Undecaprenyldiphospho‐muramoylpentapeptide beta‐N‐acetylglucosaminyltransferase	J5O10_13805	*								
		UDP‐N‐acetylglucosamine 1‐carboxyvinyltransferase	J5O10_05030						*		*	
		N‐acetylmuramic acid 6‐phosphate etherase	J5O10_15565									*
		Anhydro‐N‐acetylmuramic acid kinase anmK	J5O10_15590									*
		N‐acetylmuramoyl‐l‐alanine amidase AmiA	J5O10_14545		*		*				*	*
		Alanine racemase	J5O10_17565	*	*	*					*	*
		L%2CD‐transpeptidase/peptidoglycan binding protein	J5O10_15145	*		*					*	
		peptidoglycan DD‐metalloendopeptidase family protein	J5O10_09410				*					*
		Peptidoglycan editing factor PgeF	J5O10_13745	*		*						*
		Peptidoglycan‐binding protein	J5O10_12345	*		*						*
Germination		Spore cortex‐lytic germination protein SleC	J5O10_02975	*		*					*	*
		Bile acid germinant receptor pseudoprotease CspC	J5O10_11655	*		*						*
		Bifunctional germination protease/germinant receptor pseudoprotease CspBA	J5O10_11660			*						*
Virulence genes		ADP‐ribosylating binary toxin enzymatic subunit CdtA	J5O10_13575									*
		ADP‐ribosylating binary toxin enzymatic subunit CdtB	J5O10_13580	*								*
		Glycosylating toxin TcdA	J5O10_03485	*	*	*	*					*
		Glycosylating toxin TcdB	J5O10_03470	*	*	*	*					*
		TcdC	J5O10_03490									
		Holin‐like glycosylating toxin export protein TcdE	J5O10_03475									
		Glycosylating toxin sigma factor TcdR	J5O10_03465									
Motility		Flagellar motor protein	J5O10_03915			*						
		Isopeptide‐forming pilin‐related protein SpaA	J5O10_09435		*							*
Ethanolamine synthesis		Ethanolamine ammonia‐lyase reactivating factor EutA	J5O10_09865	*		*	*					
		Ethanolamine ammonia‐lyase subunit EutB	J5O10_09870	*		*	*					*
		Ethanolamine ammonia‐lyase activity EutC	J5O10_09875	*		*	*					*
		Ethanolamine utilization phosphate acetyltransferase EutD	J5O10_09905	*			*					
		Ethanolamine utilization protein EutH	J5O10_09925	*		*						*
		Ethanolamine utilization protein EutH	J5O10_03900									*
		Ethanolamine utilization microcompartment protein EutL	J5O10_09880	*		*						*
		Ethanolamine carboxysome structural protein EutM	J5O10_09885	*		*						*
		CcmL family microcompartment protein EutN	J5O10_09915	*		*						
		Ethanolamine utilization protein EutQ	J5O10_09930	*		*						*
		Ethanolamine utilization protein EutS	J5O10_09845	*		*						*
		Ethanolamine utilization cobalamin adenosyltransferase EutT	J5O10_09900	*		*						*
		TIGR02536 family ethanolamine utilization protein	J5O10_09910	*		*						
		Acetaldehyde dehydrogenase (acetylating) EutE	J5O10_09890	*		*	*		*			*
Ornithine synthesis	Acetylglutamate kinase		J5O10_10550							*		*
	N‐acetyl‐gamma‐glutamyl‐phosphate reductase		J5O10_10560			*		*		*		*
	Ornithine carbamoyl transferase		J5O10_10540									*
	GlmL‐related ornithine degradation protein		J5O10_02420									*
	d‐ornithine 4%2C5‐aminomutase subunit		J5O10_02415									*
	ornithine aminomutase subunit alpha		J5O10_02410									*
	2%2C4‐diaminopentanoate dehydrogenase		J5O10_02415					*				*
	2‐amino‐4‐oxopentanoate thiolase subunit		J5O10_02400									*
	2‐amino‐4‐oxopentanoate thiolase subunit		J5O10_02405									*
Rnf system	RnfABCDGE type electron transport complex subunit B		J5O10_05470	*		*					*	*
	RnfABCDGE type electron transport complex subunit D		J5O10_05450	*		*					*	*
	electron transport complex subunit E		J5O10_05460	*		*						*
	RnfABCDGE type electron transport complex subunit G		J5O10_05455	*		*					*	*
	Electron transport complex subunit RsxA		J5O10_05465	*		*	*		*		*	*
Flavoprotein	Electron transfer flavoprotein subunit beta EtfA		J5O10_02245	*		*						*
	Electron transfer flavoprotein subunit alpha/FixB family protein EtfB		J5O10_02240	*		*						*
	Flavodoxin family protein		J5O10_02355									*
	Flavodoxin family protein		J5O10_04210									*
	Flavodoxin family protein		J5O10_14750									*
	Electron transfer flavoprotein subunit beta/FixA family protein		J5O10_05125				*				*	
	Electron transfer flavoprotein subunit alpha/FixB family protein		J5O10_05130				*					*
	FprA family A‐type flavoprotein		J5O10_05545		*							
	Electron transfer flavoprotein subunit beta/FixA family protein		J5O10_05615				*					*
	Electron transfer flavoprotein subunit alpha		J5O10_05620				*					*
	Bifunctional riboflavin kinase/FAD synthetase		J5O10_06360									*
	FprA family A‐type flavoprotein		J5O10_08050	*		*					*	*
	Riboflavin synthase		J5O10_08410									*
	Flavodoxin		J5O10_10430		*							
	Flavin reductase family protein		J5O10_10990	*		*						*
	Flavodoxin domain‐containing protein		J5O10_11490									*
	Flavin reductase		J5O10_12385								*	*
	NADH:flavin oxidoreductase		J5O10_14755									*
Iron metabolism	desulfoferrodoxin family protein		J5O10_04290		*				*			*
	EFR1 family ferredoxin		J5O10_15985	*								
	Ferritin		J5O10_11425									*
	Ferrous iron transport protein A		J5O10_07185	*								
	Ferrous iron transport protein A		J5O10_07190	*	*	*						*
	Ferrous iron transport protein A		J5O10_07425	*		*					*	*
	Ferrous iron transport protein A		J5O10_08710		*		*					
	Ferrous iron transport protein B		J5O10_07195		*	*	*		*			
	Ferrous iron transport protein B		J5O10_07420	*		*					*	*
	Ferrous iron transport protein B		J5O10_16770			*					*	
	Ferric uptake regulation protein Fur		J5O10_04290		*				*			*
	Hydroxylamine reductase		J5O10_11285			*						
	Indolepyruvate ferredoxin oxidoreductase subunit alpha IorA		J5O10_12285	*		*						*
	Iron ABC transporter permease		J5O10_08600									*
	Iron ABC transporter permease		J5O10_16040	*		*						*
	Iron hydrogenase		J5O10_16695	*		*						*
	Iron‐containing alcohol dehydrogenase		J5O10_15865									*
	Iron‐only hydrogenase system regulator		J5O10_11210				*		*		*	
	NifB/NifX family molybdenum‐iron cluster‐binding protein		J5O10_08385		*							
	NifH nitrogenase iron protein NifH		J5O10_08590				*			*		*
	30S ribosomal protein S12 methylthiotransferase RimO		J5O10_06415				*		*		*	
	Phosphomethylpyrimidine synthase ThiC		J5O10_08435				*					*

From 0 to 5		From 0 to −4.99	
From 5 to 10		From −5‐ to −9.99	
From 10 to 15		From −10 to −14.99	
From 15 to 30		From −15 to −30	

#### 3.3.2 Effects of the presence of two different microbiota on *C. difficile* gene expression

At the 12 h time point, the expression of several genes was downregulated in the samples with microbiota present compared to the control samples (CT‐FD1 = 648 and CT‐FD2 = 197) (Figure 4b). At the 24‐h time point, there were fewer significant differences in gene expression in samples with microbiota (Figure 4c). These genes were studied based on the 14 selected categories. The detailed gene expression data are shown in Appendix A: Table A3.

**Figure 5:**
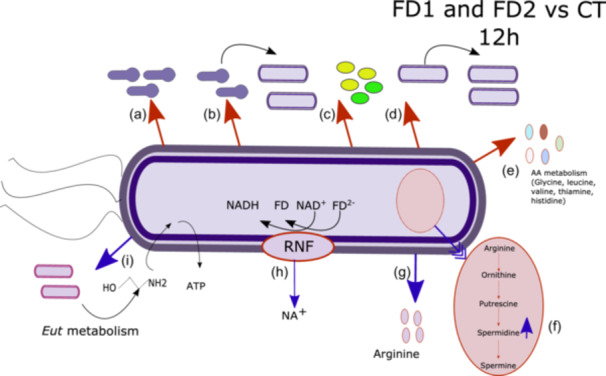
Impact of bacterial microbiota on gene expression of *Clostridioides difficile* at 12 h. A schematic representation of the Analysis 1: bacterial microbiota effect on gene expression of *C. difficile*. Different categories of genes are represented in this figure: (a) Genes associated with sporulation, (b) germination‐associated genes, (c) genes associated with toxin production, (d) genes associated with cell growth, (e) genes associated with amino acid, (f), genes associated with spermidine metabolism, (g) genes associated with arginine metabolism, (h) Rnf complex associated genes, (i) genes involved in ethanolamine metabolism. Red arrow: the gene expression is higher in the FD conditions compared to the control and blue arrow: the gene expression is lower in the FD conditions compared to the control.

At the 12 h time point, compared with those in control (CT), the expression of genes involved in sporulation, germination, virulence, cell division and iron metabolism significantly decreased in the presence of the microbiota (see Table 1). In the presence of the microbiota, the expression of genes coding for sporulation and other regulators (*sigH*, *spo0A, rstA*, and *codY*) was significantly decreased. In the presence of the microbiota, the gene expression of *tcdA* and *tcdB* was significantly downregulated compared to the control group. In the presence of the microbiota, the gene expression of *sleC* and *cspC* was significantly downregulated compared to the control. In the presence of the microbiota, the gene expression of *minE* was significantly downregulated compared to the control. In the presence of the microbiota, the gene expression of ferrous iron transport proteins A and B was significantly downregulated compared to the control. The expression of the permease subunit of the iron chelate uptake ABC transporter family was downregulated at 12 h and upregulated at 24 h compared to that in the control group. Figure 5 illustrates the impact of microbiota on gene expression of *C. difficile* at 12 h.

At the 12 h time point, compared with those in control (CT), a significant increase in the expression of genes encoding the Rnf system, ethanolamine metabolism and cell wall binding proteins was observed (see Table 1). In the presence of the microbiota, the gene expression of *rnfBDEG* and hydroxyisocaproate CoA‐transferase was significantly upregulated compared to that in the control group. In the presence of the microbiota, the gene expression of 13 genes involved in ethanolamine metabolism was significantly upregulated compared to that in the control group (see Figure 5). In the presence of the microbiota, the gene expression of *cpw7* and *cpw22* was significantly upregulated compared to that in the control group.

At the 24‐h time point, compared with those in control (CT), the expression of genes involved in sporulation (FD2), virulence, cell division, and cell wall‐binding proteins significantly decreased in the presence of the microbiota (see Table 1). In the presence of the microbiota, the gene expression of *minE* was significantly downregulated compared to the control. In the presence of the microbiota, the gene expression of *tcdA* and *tcdB* was significantly downregulated compared to that in the control group. In the presence of the microbiota, the gene expression of *cpw9*, *cpw22*, and *cpw84* was significantly downregulated compared to that in the control group. In the presence of donor 2 (FD2), the expression of genes coding for sporulation (*cotJA*, *spoIIR*, *spoIIAG*, *spoIIIAH*, and *YabG*) decreased significantly.

At the 24‐h time point, compared with those in control (CT), the expression of genes involved in sporulation (FD2), and iron metabolism significantly increased in the presence of the microbiota (see Table 1). In the presence of the microbiota, the gene expression of ferrous iron transport proteins A and B was significantly upregulated compared to the control. In the presence of Donor 2 (FD2), the expression of *sigH* was significantly upregulated compared to the control.

The KEGG modules and KO numbers were obtained using GhostKOALA. The results are shown in Table 2. Supporting Information: Table S4 lists CDSs for which a KEGG module or KO annotation is available and for which DESEQ2 analysis showed a significant difference in abundance (https://zenodo.org/doi/10.5281/zenodo.13121217). In the presence of the microbiota, a decrease in the expression of genes involved in amino acid metabolism was observed at 12 h. A decrease in the expression of genes coding for sporulation and cell division was observed, and an increase in the expression of genes coding for secondary metabolism (spermidine and ethanolamine) was shown. A decrease in the expression of genes encoding genes involved in amino acid metabolism was observed at 24 h. In Donor 2 (FD2), an increase in the expression of genes involved in secondary metabolism (ornithine, polyamine, and ethanolamine) was observed compared to that in the control.

**Table 2:** Microbiota effect and sampling time effect on *Clostridioides difficile* metabolism (KEGG modules and KO numbers).

Time	Conditions	Number of modules	Modules	KO numbers	KO functional orthologs
Microbial context					
12 h (*n* = 6)	FD1 vs. CT AND FD2 vs. CT	14	↓ Valine/isoleucine biosynthesis, pyruvate => valine/2‐oxobutanoate => isoleucine ↓ Histidine biosynthesis, PRPP ↓ Leucine biosynthesis, 2‐oxoisovalerate => 2‐oxoisocaproate ↓ Isoleucine biosynthesis, pyruvate => 2‐oxobutanoate ↓ Thiamine biosynthesis, archaea, AIR (+NAD+) => TMP/TPP	128	10 KO ↓ Sporulation, 2 KO ↓ Cell division, 2 KO ↓ and 1 KO ↑ Iron metabolism, 4 KO ↓ glycine metabolism, 1 KO ↓ virulence, 1 KO ↑ spermidine, 10 KO ↑ Ethanolamine.
	FD2 vs. CT	17	↓ Serine biosynthesis, glycerate‐3P => serine	83	3 KO ↓ Sporulation, 2 KO ↑ spermidine, 2 KO ↑ quorum sensing.
	FD2 vs. CT	14	↓ Isoleucine biosynthesis, threonine => 2‐oxobutanoate => isoleucine	71	7 KO ↓ Sporulation, 2 KO ↓ Cell division, 2 KO ↓ Cellobiose, 5 KO ↑ proline metabolism.
24 h (*n* = 3)	FD2 vs. CT AND FD1 vs. CT	11	↓ Cysteine biosynthesis, serine	79	4 KO ↓ AA metabolism, 1 KO ↑ virulence, 2 KO ↓ Cell division.
	FD2 vs. CT	43	↑ Arginine biosynthesis, ornithine => arginine ↑ Arginine biosynthesis, glutamate => acetylcitrulline => arginine ↑ Valine/isoleucine biosynthesis, pyruvate => valine/2‐oxobutanoate => isoleucine ↑ Ornithine biosynthesis, glutamate => ornithine ↑ Thiamine biosynthesis, prokaryotes, AIR (+DXP/tyrosine) => TMP/TPP ↑ Thiamine biosynthesis, prokaryotes, AIR (+DXP/glycine) => TMP/TPP ↑ Polyamine biosynthesis, arginine => agmatine => putrescine => spermidine ↑ Pyrimidine ribonucleotide biosynthesis, UMP = > UDP/UTP, CDP/CTP ↑ De novo pyrimidine biosynthesis, glutamine (+PRPP) => UMP ↑ Pyrimidine deoxyribonucleotide biosynthesis, UDP => dTTP ↑ Tyrosine biosynthesis, chorismate => HPP => tyrosine ↓ Tyrosine biosynthesis, chorismate => HPP => tyrosine ↓ Isoleucine biosynthesis, threonine => 2‐oxobutanoate => isoleucine ↓ Phenylalanine biosynthesis, chorismate => phenylpyruvate => phenylalanine	82	2 KO ↑ Sporulation (stage IV), 7 KO ↓ Sporulation, 3 KO ↑ proline metabolism, 2 KO ↑ glycine metabolism, 3 KO ↑ Ethanolamine.
	FD1 vs. CT	16	↓ Serine biosynthesis, glycerate‐3P => serine ↓ Histidine biosynthesis, PRPP => histidine ↓ Fatty acid biosynthesis, initiation	113	3 KO ↓ Flavodoxin, 1 KO ↑ Iron metabolism.
Time	Conditions	Number of modules	Modules	KO numbers	KO functional orthologs
Time context					
24 h vs. 12 h (*n* = 3)	FD1 and FD2	10	↓ Cysteine biosynthesis, methionine => cysteine ↓ Methionine degradation	133	7 KO ↑ Sporulation 4 KO ↓ Cell division 5 KO ↑ proline metabolism 1 KO ↑ and 1 KO ↓ glycine metabolism 5 KO ↓ Rnf system
	FD2	11	↓ Cysteine biosynthesis, serine => cysteine ↓ Thiamine biosynthesis, prokaryotes, AIR (+DXP/tyrosine) => TMP/TPP	56	2 KO ↓ Cell division 2 KO ↓ Sporulation 3 KO ↓ Flavodoxin
	FD1	40	↓ Arginine biosynthesis, glutamate => acetylcitrulline => arginine ↓ Phenylalanine biosynthesis, chorismate => phenylpyruvate => phenylalanine ↑ Polyamine biosynthesis, arginine => agmatine => putrescine => spermidine ↑ Fatty acid biosynthesis, elongation ↑ Thiamine biosynthesis, archaea, AIR (+NAD+) => TMP/TPP	401	20 KO ↑ Sporulation 1 KO ↑ and 7 KO ↓ Ethanolamine 4 KO ↑ Ornithine metabolism

Abbreviations: CT, control; KEGG, Kyoto Encyclopedia of Genes and Genomes; KO, KEGG orthology.

Differences in gene expression were observed between the two fecal donor groups. At 12 h, the expression of genes encoding the cellobiose PTS was significantly downregulated in FD1 and significantly upregulated in FD2. The expression of genes involved in proline metabolism (*prd*) was significantly upregulated in FD1 at 12 h and in FD2 at 24 h. The expression of genes involved in ornithine metabolism increased in FD2 at 24 h. The expression of antimicrobial resistance genes *(arg* genes) was significantly upregulated in FD2. The expression of genes encoding spermidine and putrescine transport systems (*pot* genes) was significantly upregulated in FD2 at 12 h. The expression of genes encoding d‐glucosaminate PTS (*dga*) genes was significantly downregulated in FD1 at 12 h. The expression of genes encoding the *clp* gene was upregulated in FD2 at 24 h and in FD1 at 12 h and was downregulated in FD2 at 12 h. The main significant differences in terms of *C. difficile* gene expression between the two different microbiota are listed in Table A4 (Appendix A). The microbial profiles of the two donors were described in a preliminary study (Martinez et al., 2022).

#### 3.3.3 Effect of incubation time on *C. difficile* gene expression

For each condition, the data from the 24‐h and 12‐h analyzes were compared to identify differences. There were many differences in gene expression between these two time points in FD1 (n = 1075, of which 658 were overexpressed) and FD2 (n = 307, of which 107 were overexpressed) (Figure 4a).

According to the results of the KEGG module and KO number analyzes, there was a decrease in the expression of genes involved in amino acid metabolism (see Table 2). An increase in the expression of genes involved in ornithine metabolism, sporulation and proline metabolism was observed at 24 h. Between 12 h and 24 h, gene expression of ornithine metabolism was increased in the control (CT).

Between 12 h and 24 h, compared with those in control (CT), the expression of genes involved in sporulation, germination, virulence, ornithine metabolism, and iron metabolism was significantly increased in the presence of the microbiota (see Table 1). In the presence of the microbiota, the gene expression of the ferrous iron transport proteins A and B was significantly upregulated at 24 h compared to 12 h. In the presence of donor 1 (FD1), the expression of genes coding for sporulation stage II, stage III and stage IV was significantly increased at 24 h compared to 12 h. In the presence of donor 1 (FD1), the gene expression of *cdtA*, *cdtB*, *tcdA* and *tcdB* was significantly upregulated at 24 h compared to 12 h. In the presence of donor 1 (FD1), the gene expression of *sleC*, *cspC* and *CspBA* was significantly upregulated at 24 h compared to 12 h. In the presence of donor 1 (FD1), the gene expression of genes associated with ornithine metabolism (*oraE*, *oraS*, *ortA*, and *ortB*) was significantly upregulated at 24 h compared to 12 h.

Between 12 h and 24 h, compared with those in control (CT), the expression of genes involved in the Rnf system, cell wall binding proteins, ethanolamine, and cell division metabolism was significantly decreased in the presence of the microbiota (see Table 1). In the presence of the microbiota, the gene expression of *minE*, *rodA*, *sepF*, and *ftsZ* was significantly downregulated compared to the control. In the presence of the microbiota, the gene expression of *rnfBDG* was significantly downregulated at 24 h compared to 12 h. In the presence of the microbiota, the gene expression of *cpw19*, *cpw22*, and *cpw84* was significantly downregulated compared to that in the control group. In the presence of donor 1 (FD1), the gene expression of 10 genes involved in ethanolamine metabolism was significantly downregulated at 24 h compared to 12 h.

### 3.4 Study of *C. difficile* virulence and metabolism

**Figure 6:**
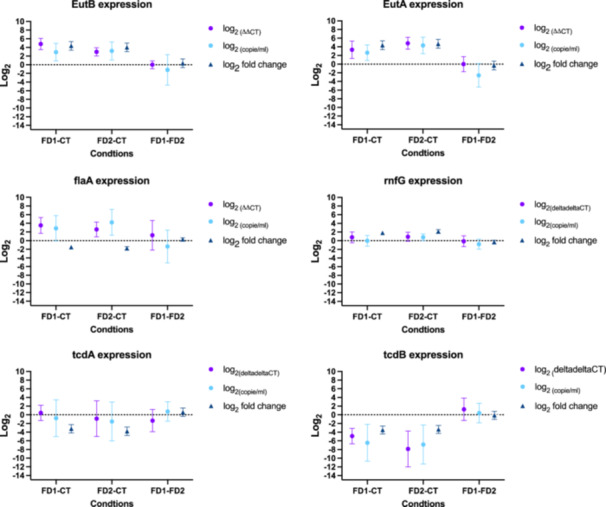
Validation of RNAseq data using quantitative polymerase chain reaction diluted 10x analysis at 12 h. (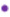) Data from qPCR using 2^−(△△CT)^ where the genes of interest are *tcdA*, *tdcB*, *flaA*, *rnfG*, *eutA*, and *eutB* and the housekeeping gene is *gluD* (Livak & Schmittgen, 2001). The FD1 and FD2 samples are the interest samples. The second element of the conditions is the control condition (CT samples or FD2 samples). To standardize the comparison in the graph, a log_2_ was applied in all the samples. (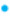) The second method is the analysis of log_2_ (interest gene copies mL^−1^) normalized with *gluD*. To standardize the comparison in the graph, a log_2_ was applied in all the samples. (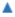) Results from DESeq2 analysis on RNAseq raw reads showed in log_2_ fold change.

The validation of the RNA‐Seq data was performed using qPCR analysis of the 6 genes (see Appendix C: Table C1): *tcdA*, *tdcB*, *flaA*, *rnfG*, *eutA*, and *eutB* at 12 h (see Figure 6 **)**. Three out of the 6 genes (*rnfG*, *eutA*, and *eutB*) displayed similar results between qPCR and RNA‐Seq. The *tcdB* and *tcdA* genes showed a similar trend, but the qPCR results had a large standard deviation. The *flaA* gene exhibited opposite results according to the RNA‐Seq, with a large standard deviation.

## 4 DISCUSSION

An in vitro coculture model was established to study the effect of the gut microbiota on *C. difficile* gene expression under anaerobic conditions. Two fecal donors were used to mimic the gut microbiota (FD1 and FD2). Several parameters were analyzed, namely, the growth of *C. difficile* under different conditions, gene expression according to RNA‐Seq and confirmation data based on RT‐qPCR. This work aimed to analyze the influence of the microbiota on *C. difficile* gene expression using a coculture model.

*C. difficile* growth was similar in the three conditions tested (see Figure 1). The presence of the microbiota had no significant positive or negative effect on the *C. difficile* growth. Under the control condition (CT), the levels of *C. difficile* were significantly increased by 4.3 log_10_ (CFU mL^−1^) after 24 h. In the same media, the growth potential in this study is doubled after 24 h compared to previous work (Martinez et al., 2022). The difference with this previous work was the volume of media in contact with *C. difficile* spores (30 mL vs. 2 mL in this study).

Sporulation is essential for *C. difficile* survival under aerobic conditions and to facilitate transmission of the bacteria. In vivo, in mice, *C. difficile* spores were detected in the colon at 24 h, and ± 20% of viable *C. difficile* spores were present (Koenigsknecht et al., 2015; Shen, 2015). The expression of genes encoding sporulation started to increase at 14 h in vivo (Janoir et al., 2013). The rate of germination depends on optimum conditions such as pH, anaerobiosis, presence of germinants and temperature (Wheeldon et al., 2008). The genes associated with germination are *sleC*, *cspBA*, and *cspC*. *cspBA* is expressed as a fusion protein during sporulation and is cleaved by the YabG protease into two proteins, CspB and CspA. CspC and CspA are pseudoproteases that recognize germinants in the external environment (Francis et al., 2013). SleC *is* a key lytic transglycosylase that is involved in spore germination (Gutelius et al., 2014). In this study, in the presence of microbiota, the expression of genes associated with germination, sporulation and other regulators (*sigH*, *spo0A*, *rstA*, and *codY*) was significantly reduced compared to the control. In the presence of feces from donor 2 (FD2), the expression of *sigH* was decreased and the expression of *spo0A* was increased compared to the control (CT). In a study in vitro, expression of the *spo0A* was downregulated in the stationary phase, while expression of several genes involved in sporulation was upregulated in the stationary phase in the casamino acid medium (Hofmann et al., 2018). In the presence of feces from donor 1 (FD1), the expression of genes involved in sporulation and germination was significantly upregulated at 24 h compared to 12 h. These results showed that the microbiota influences *C. difficile* gene expression by reducing genes associated with sporulation and germination. This study didn't show the presence of germination and sporulation, but the growth curve showed that there was growth of the vegetative forms, so spore germination did occur in this study. One hypothesis for this reduction is that essential nutrients for germination (proline and taurocholic acid) are less available due to competition from other bacteria (Reed & Theriot, 2021).

Toxins are essential for *C. difficile* pathology. The genes present in PaLoc are *tcdR*, *tcdB*, *tcdE*, *tcdA*, and *tcdC*. *spo0A* negatively regulates toxin genes (Deakin et al., 2012) in ribotype 027 (Edwards & McBride, 2014), but this regulation occurs during the early stationary phase in ribotype 078 (Edwards & McBride, 2014; Mackin et al., 2013). The presence of microbiota has a negative impact on gene expression associated with toxins genes (*tcdA* and *tcdB*). In the presence of feces from donor 1 (FD1), the gene expression of *tcdA*, *tcdB*, *cdtA*, and *cdtB* was upregulated at 24 h compared to 12 h. In vivo, mice exhibited symptoms 6 h after toxin detection (Shen, 2015). In vitro and in vivo, the gene expression of *tcdA* and *tcdB* increased after 38 h in mice, in TY medium and *C. difficile* 630 (Janoir et al., 2013), and in the stationary phase in casamino acid medium in *C. difficile* 630△*erm* (Hofmann et al., 2018). Toxin synthesis is correlated with nutrient limitation (Chandra, 2022; Dineen et al., 2007). The presence of glucose (Dupuy & Sonenshein, 1998), cysteine (Karlsson et al., 2008), proline (Karlsson et al., 1999) and glycine (Karlsson et al., 1999) decreases toxin production. This study did not measure the toxins levels. One hypothesis of this reduction of gene expression is that fewer nutrients were available for germination and thus the production of toxins.

The Stickland pathways use amino acids as an energy source (Neumann‐Schaal et al., 2019). The genes involved in the reductive pathway are hydroxyisocaproate CoA‐transferase (*hadAIBC*), acetyl‐CoA dehydrogenase (*acdB*), and electron transfer flavoprotein subunit beta and alpha (*etfBA*) and the genes involved in the oxidative pathway are ferredoxins (4S‐4F ferredoxins) (Supporting Information: Table S2: https://zenodo.org/doi/10.5281/zenodo.13121217). In the presence of microbiota, expression of genes involved in amino acid metabolism (valine, histidine, leucine, isoleucine, cysteine) was decreased. In the presence of the microbiota, the expression of genes encoding hydroxyisocaproate CoA‐transferase and the Rnf complex was significantly upregulated. In donor 1 (FD1), the expression of genes involved in ornithine metabolism was increased at 24 h compared to 12 h. In vitro, expression of the *prd* and *grd* operons was also increased in the early stationary phase (Hofmann et al., 2018), and the expression of genes involved in cysteine metabolism (*cysE*, *cysK*, and J5010_007910) increased in the transient phase. In casamino acid media, the expression of genes in the Rnf complex decreased in the late stationary phase (Hofmann et al., 2018). In this study, both Stickland metabolism pathways were activated in *C. difficile* transcriptome analysis in the presence of the microbiota.

Another source of nutrients in the intestine is ethanolamine, which is a ubiquitous component of the cell membrane (Nawrocki et al., 2018). This nutrient is a source of carbon and nitrogen (Nawrocki et al., 2018). The use of ethanolamine is advantageous for bacteria in nutrient‐limited environments. Ethanolamine utilization is controlled by the *eut* operon which consists of 19 genes (Nawrocki et al., 2018). Throughout the growth phase, the expression of genes coding for ethanolamine metabolism was significantly upregulated compared to that in the control group. At 24 h, the expression of 11 genes was downregulated in the presence of both microbiota. This can be explained by the presence of eucaryotic cells in human feces (FD1 and FD2).

Iron is a key element in many metabolic and cellular pathways (Miethke & Marahiel, 2007). The use of iron by pathogenic bacteria is beneficial. The major transcriptional repressor is Fur (a ferric uptake regulator) (Ho & Ellermeier, 2015; Troxell & Hassan, 2013). In an iron‐limited environment, Fur activates several genes involved in iron acquisition systems (Ho & Ellermeier, 2015), and Fur inactivation allows the expression of genes (Berges et al., 2018). Fur also regulates ferredoxin genes and genes associated with flavoproteins (Ho & Ellermeier, 2015). In this study, the gene expression of *C. difficile* genes associated with iron metabolism suggested that the environment was deficient in iron. In the gastrointestinal tract, iron is limited because bacteria and the host compete for iron (Skaar, 2010). In this study, the expression of genes encoding the Rnf system significantly increased in the presence of microbiota. At 12 h, the expression of *etfA* and *etfB* was significantly greater than that in the control group. At 24 h, the expression of the FprA family A‐type flavoprotein, flavin reductase family protein and NADH:flavin oxidoreductase significantly decreased compared to that in the control group. In the literature, under an iron‐limited environment, the expression of genes encoding other metabolic pathways, such as flavodoxin (FldX), proline reductase (Prd), the iron pump ion Rnf complex and the reaction 5‐aminovalerate, was upregulated and the expression of genes encoding ferredoxin‐dependent amino acid fermentation products was downregulated (*had*, *etf*, *acd*, *grd*, *trx*, *bdc*, and *hbd*) (Berges et al., 2018; Ho & Ellermeier, 2015). In vitro, the expression of *etfAB* increased throughout the growth phase (Hofmann et al., 2018). Based on the expression of these genes, one hypothesis is that this environment at 12 h was iron‐limited by the presence of the microbiota and that *C. difficile* established a number of metabolic pathways to overcome this iron limitation.

Differences in gene expression were observed between the two fecal donor groups. At 12 h, the expression of genes encoding the cellobiose PTS was significantly downregulated in FD1 and significantly upregulated in FD2. In the literature, the *cel* operon allows *C. difficile* to use cellobiose as a carbon source in a depleted environment (Hasan et al., 2021). Cellobiose metabolism is linked to sporulation (Hasan et al., 2021). Indeed, a *celA* mutant produces fewer spores than a wild‐type (Hasan et al., 2021). *C. difficile* detected the cellobiose in the environment and activated genes in the *cel* operon. This suggests that cellobiose is present in the environment in our transwell model when feces are present. The hypothesis is that cellobiose is present in the environment and originates the degradation of the cellulose into oligosaccharides and cellobiose by the bacteria (*Cellulomonas* spp., *Bacteroides* spp., *Clostridium* spp., *Ruminococcaceae*, and *Streptomyces* spp.) (Koeck et al., 2014; Parisutham et al., 2017). The bacterial profiles of two fecal donors (FD1 and FD2) in our study showed that the relative abundance of *Clostridiales*_ge, *Ruminococcaceae* and *Bacteroides* was 20.9% and 7.3% for FD2 and FD1, respectively. The proportion of these bacteria is higher in FD2 suggesting that cellobiose is more present in the presence of FD2. This needs to be confirmed by determining the concentration of cellobiose in the inner and outer compartments.

The expression of genes involved in proline metabolism (*prd*) was significantly upregulated in FD1 at 12 h and in FD2 at 24 h in comparison with the control (CT). The expression of genes involved in ornithine metabolism increased in FD2 at 24 h. Ornithine is a key nutrient that allows *C. difficile* to colonize the asymptomatic gut (Marshall et al., 2023). Proline inhibits the production of the toxin (Karlsson et al., 1999). The ability to utilize ornithine (ornithine transcarbamylase) has been observed in other bacteria: *Escherichia coli*, *Mycobacterium tuberculosis*, *Pyrococcus furiosus*, *Thermotoga maritima*, *Pseudomonas aeruginosa*, and *Thermus thermophilus* (Shi et al., 2015). A hypothesis is suggested to explain the differential expression of ornithine and proline metabolism: proline is directly available and utilized in the FD1 microbiota at 12 h and several steps are realized to metabolize proline in the FD2 microbiota at 24 h.

The expression of genes encoding spermidine and putrescine was significantly upregulated in FD2 at 12 h. These are polyamines (Emerson et al., 2008). Putrescine is produced from arginine by arginine decarboxylase or from ornithine by ornithine decarboxylase (Emerson et al., 2008). Many food‐borne bacteria are capable of producing biogenic amines: *Staphylococcus* spp., *Bacillus* spp., lactic acid bacteria (*Lactobacillus* spp., *Pediococcus* spp., *Enterococcus* spp.), *Pseudomonas* spp., and a lot of *Enterobacteriaceae* (*Salmonella* spp., *Proteus* spp., *Serratia* spp., *Yersinia* spp., *Citrobacter* spp., *Enterobacter* spp., *Escherichia* spp., and *Klebsiella* spp,…) (Wunderlichová et al., 2014). The bacterial profiles of two fecal donors in our study showed that the relative abundance of these bacteria is not relevant (<1.0%).

The expression of antimicrobial resistance genes (*arg* genes) was significantly upregulated in FD2. The expression of genes encoding d‐glucosaminate PTS (*dga*) genes was significantly downregulated in FD1 at 12 h. The expression of genes encoding *clp* genes was upregulated in FD2 at 24 h (*clpB*) and in FD1 at 12 h (*clpB*) and downregulated in FD2 at 12 h (*clpX* and *clpP*). *clpB* expression is upregulated with heat stress in *C. difficile* (Jain et al., 2011; Ternan et al., 2014).

qPCR analysis was used to compare and confirm the RNA‐Seq data. According to the literature, between 15.1% and 19.4% of genes exhibit dissimilarity between qPCR and RNA‐Seq results. Our results indicate a similar concordance; with 83.33% of the studied genes showing similarities with RNA‐Seq.

An interesting aspect of this work is the investigation of the gene expression of a *C. difficile* strain belonging to PCR‐ribotype 078. This PCR‐ribotype was chosen because it is one of the five most common PCR‐ribotypes in Belgium (Callies et al., 2024), contains both virulence operons (PaLoc and CdtLoc) and is widespread in animals (Rodriguez et al., 2012). Several perspectives of this work are (i) studying other relevant PCR‐ribotypes (such as 014 and 027) to validate this effect of microbiota; (ii) increasing the volume of the inner compartment (75 mm Transwell [VWR]) and quantifying several phenotypes (such as SCFA, PBS, SBS, l‐proline, l‐ornithine, and toxins); (iii) using mutants (such as a deletion of *tcdA*, *tcdB*, *cdtA*, and *cdtB*) to see the effect of these proteins on *C. difficile* growth in contact with microbiota.

To study the impact of the microbial community, we used samples from two fecal donors. Further work is needed to determine the differences between fecal donors. The perspectives of this work for studying the impact of microbiota on *C. difficile* gene expression are (i) to develop metatranscriptomic analysis in the outer compartment; and (ii) to simplify the outer compartment by using a bacterial consortium.

In conclusion, the presence of microbiota did not affect the *C. difficile* growth in this study. The presence of microbiota did affect the expression of *C. difficile* genes such as sporulation genes, germination genes and virulence genes. These three categories of genes are essential for the transmission of the pathology. In the presence of microbiota, *C. difficile* exerts a defence mechanism to survive the competition (iron‐limited environment and ethanolamine metabolism). To further investigate this interaction, future studies will use a simplified coculture model with an artificial bacterial consortium, replacing the use of fecal samples.

## AUTHOR CONTRIBUTIONS

**Elisa Martinez:** Conceptualization; data curation; formal analysis; visualization; writing—original draft; methodology; investigation; software; funding acquisition; writing—review and editing. **Noémie Berg**: Conceptualization; formal analysis; data curation; software. **Cristina Rodriguez**: Methodology; supervision; writing—review and editing. **Georges Daube**: Supervision; investigation; project administration; writing—review and editing; validation; funding acquisition; resources; methodology. **Bernard Taminiau**: Software; validation; funding acquisition; resources; methodology; investigation; supervision; writing—review and editing; writing—original draft; data curation; formal analysis.

## CONFLICT OF INTEREST STATEMENT

The authors declare no conflicts of interest.

## ETHICS STATEMENT

The study was conducted according to the guidelines of the Declaration of Helsinki and approved by the Ethics Committee of the Hospital‐Faculty Ethics Committee of the University of Liège, Belgium (2016/331‐BE7072016306919, 2016). Written informed consent was obtained from all the participants involved in the study.

## Supporting information

Supporting information.

Supporting information.

Supporting information.

Supporting information.

Supporting information.

## Data Availability

The data sets generated during the current study are openly available in the NCBI database under accession numbers PRJNA1023484 (RNA sequencing): https://www.ncbi.nlm.nih.gov/bioproject/PRJNA1023484 and PRJNA716140 (DNA sequencing): https://www.ncbi.nlm.nih.gov/bioproject/PRJNA716140. All data are provided in full in this article except for the data in Table S1, Table S2, Table S3, and Table S4 which are available in the Zenodo repository at https://zenodo.org/doi/10.5281/zenodo.13121217.

## APPENDIX A

**Table A1:** Corresponding SRA metadata.

Experiment	Library ID in GenBank	Library ID in paper
1	Insert2‐T12‐replicates‐1_NGS22‐ W265_AH7TV2DSX3_S51_L001	CT – 12 h – replicate 1
1	Insert2‐T12‐replicates‐2_NGS22‐ W266_AH7TV2DSX3_S52_L001	CT – 12 h – replicate 2
1	Insert2‐T12‐replicates‐3_NGS22‐ W267_AH7TV2DSX3_S53_L001	CT – 12 h – replicate 3
1	Insert3‐T12‐replicates‐1_NGS22‐ W268_AH7TV2DSX3_S54_L001	FD1 – 12 h – replicate 1
1	Insert3‐T12‐replicates‐2_NGS22‐ W269_AH7TV2DSX3_S55_L001	FD1 – 12 h – replicate 2
1	Insert3‐T12‐replicates‐3_NGS22‐ W270_AH7TV2DSX3_S56_L001	FD1 – 12 h – replicate 3
1	Insert4‐T12‐replicates‐1_NGS22‐ W271_AH7TV2DSX3_S57_L001	FD2 – 12 h – replicate 1
1	Insert4‐T12‐replicates‐2_NGS22‐ W272_AH7TV2DSX3_S58_L001	FD2 – 12 h – replicate 2
1	Insert4‐T12‐replicates‐3_NGS22‐ W273_AH7TV2DSX3_S59_L001	FD2 – 12 h – replicate 3
2	Insert‐sans‐MF‐T12h‐rep‐1‐R7_NGS22‐ W937_AHJH7CDSX3_S274_L002	CT – 12 h – replicate 1
2	Insert‐sans‐MF‐T12h‐rep‐2‐R8_NGS22‐ W938_AHJH7CDSX3_S275_L002	CT – 12h – replicate 2
2	Insert‐sans‐MF‐T12h‐rep‐3‐R9_NGS22‐ W939_AHJH7CDSX3_S276_L002	CT – 12h – replicate 3
2	Insert‐sans‐MF‐T24h‐rep‐1‐R10_NGS22‐ W940_AHJH7CDSX3_S277_L002	CT – 24 h – replicate 1
2	Insert‐sans‐MF‐T24h‐rep‐2‐R11_NGS22‐ W941_AHJH7CDSX3_S278_L002	CT – 24h – replicate 2
2	Insert‐sans‐MF‐T24h‐rep‐3‐R12_NGS22‐ W942_AHJH7CDSX3_S279_L002	CT – 24h – replicate 3
2	Insert‐2‐MF4‐T12h‐replicate‐1_NGS22‐ W943_AHJH7CDSX3_S299_L002	FD1 – 12 h – replicate 1
2	Insert‐2‐MF4‐T12h‐replicate‐2_NGS22‐ W944_AHJH7CDSX3_S300_L002	FD1 – 12h – replicate 2
2	Insert‐2‐MF4‐T12h‐replicate‐3_NGS22‐ W945_AHJH7CDSX3_S301_L002	FD1 – 12h – replicate 3
2	Insert‐2‐MF4‐T24h‐replicate‐1_NGS22‐ W945_AHJH7CDSX3_S302_L002	FD1 – 24 h – replicate 1
2	Insert‐2‐MF4‐T24h‐replicate‐2_NGS22‐ W945_AHJH7CDSX3_S303_L002	FD1 – 24h – replicate 2
2	Insert‐2‐MF4‐T24h‐replicate‐3_NGS22‐ W945_AHJH7CDSX3_S304_L002	FD1 – 24h – replicate 3
2	IP3‐12‐4_NGS22‐X612_BHJH7KDSX3_S89_L001	FD2 – 12 h – replicate 1
2	IP3‐12‐5_NGS22‐X613_BHJH7KDSX3_S90_L001	FD2 – 12 h – replicate 2
2	IP3‐12‐6_NGS22‐X614_BHJH7KDSX3_S91_L001	FD2 – 12 h – replicate 3
2	IP3‐24‐4_NGS22‐X615_BHJH7KDSX3_S92_L001	FD2 – 24 h – replicate 1
2	IP3‐24‐5_NGS22‐X616_BHJH7KDSX3_S93_L001	FD2 – 24 h – replicate 2
2	IP3‐24‐6_NGS22‐X617_BHJH7KDSX3_S94_L001	FD2 – 24 h – replicate 3

**Table A2:** General characteristics of RNAseq raw reads.

Samples	Mean QC (pg/µl)	Mean RIN	Mean % Mapping	Mean % Coverage
CT_12 h (*n* = 6)	9,799.3	5.9	98.1%	92.0%
CT_24 h (*n* = 3)	888	7.7	98.2%	93.4%
FD1_12 h (*n* = 6)	2,377.2	5.5	62.4%	71.5%
FD1_24 h (*n* = 3)	694.6	7.3	77.6%	72.3%
FD2_12 h (*n* = 6)	2,650.3	5.2	53.3%	49.2%
FD2_24h (*n* = 3)	555.3	6.9	3%	2.8%

Samples names	QC (pg/µL)	RIN	% Binning (BV‐BRC)	% mapping (Subjunc)	% genome coverage (FeatureCount)
C **samples**					
CT_1_12 h	4422	7.6	94.8% CD	**97.8%**	**90.1%**
CT_2_12h	7333	5	87.0% CD	**98.1%**	**91.4%**
CT_3_12h	4835	6.1	87.8% CD	**97.9%**	**91.5%**
CT_4_12h	23,431	5.20	66.7% CD	**97.5%**	**92.0%**
CT_5_12h	18,041	4.90	75.2% CD	**98.4%**	**93.4%**
CT_6_12h	734	6.40	87.5% CD	**98.8%**	**93.4%**
CT_1_24 h	758	7.20	65.1% CD	**97.5%**	**92.7%**
CT_2_24h	917	7.90	67.9% CD	**98.9%**	**94.4%**
CT_3_24h	989	8	64.0% CD	**98.3%**	**93.2%**
**FD1 samples**					
FD1_1_12 h	370	4.3	88.2% CD	**95,9%**	**86.0%**
FD1_2_12h	9720	5.40	86.6% CD	**96,0%**	**88.2%**
FD1_3_12h	2708	6.9	89.6% CD	**96,7%**	**89.3%**
FD1_4_12h	513	4.30	30.1% CD	**81.1%**	**72.3%**
FD1_5_12h	293	6.60	NA	**43.3%**	**39.1%**
FD1_6_12h	659	5.40	NA	**62.9%**	**54.0%**
FD1_1_24 h	979	7	35.3% CD, 84.8% BL	**42.4%**	**39.8%**
FD1_2_24h	751	7.30	39.7% CD	**95.0%**	**88.4%**
FD1_3_24h	354	7.5	35.1% CD	**95.5%**	**88.8%**
**FD2 samples**					
FD2_1_12 h	356	6.8	62.8% CD	**30.2%**	**29.4%**
FD2_2_12h	4896	4.5	NA	**85.5%**	**74.9%**
FD2_3_12h	8762	6.6	60.4% CD	**95.4%**	**88.4%**
FD2_4_12h	565	7.2	NA	**35.5%**	**33.9%**
FD2_5_12h	256	3.4	NA	**34.8%**	**33.1%**
FD2_6_12h	1067	2.7	NA	**38.6%**	**35.3%**
FD2_1_24 h	452	6.9	87.3% CD, 80% R. sp., 96.9% UC	**0.1%**	**0.1%**
FD2_2_24h	311	6.6	88.7% CD, 99.1% UC, 82.4% and 69.4% R. sp., 45.8% B. sp.	**5.8%**	**5.4%**
FD2_3_24h	903	7.1	NA	**0.8%**	**0.6%**

Abbreviations: B. sp.: *Bacteroides* sp.; BL, *Bifidobacterium longum*; CD: *Clostridioides difficile*; NA, not associated; R. sp.: *Roseburia* sp.; RIN, RNA integrity number; UC, uncultured *Collinsella*.

**Table A3:** Number of significant genes from different conditions (CT, FD1, and FD2) and different time points (12 h, 24h).

Timing		12–24h (*n* = 3)	
Condition	CT	FD1	FD2
Significant genes	42	1075	307
Overexpressed genes	17	658	107
Downregulated genes	25	417	200
Timing		12h (*n* = 3)	
Condition	CT‐FD1	CT‐FD2	FD2‐FD1
Significant genes	893	366	890
Overexpressed genes	245 (27.4%)	169	144
Downregulated genes	648 (72.6%)	197	746
Timing		12h (*n* = 6)	
Condition	CT‐FD1	CT‐FD2	FD2‐FD1
Significant genes	422	422	67
Overexpressed genes	171	138	14
Downregulated genes	234	284	53
Timing		24h (n = 3)	
Condition	CT‐FD1	CT‐FD2	FD2‐FD1
Significant genes	312	324	231
Overexpressed genes	106	146	104
Downregulated genes	206	178	127

**Table A4:** Main significant differences between the *Clostridioides difficile* transcriptome in the presence of the two bacterial microbiota.

Genes	12h		24h	
	FD2	FD1	FD2	FD1
Cellobiose PTS system	*celA* 3.24	*celB* ‐ 4.2, *celC* ‐ 3.58		
* clp* genes	*clpP* ‐ 0.92, *clpX* ‐ 1.16	*clpB* 2.87	*clpB* 5.83	
* arg* genes	*argC* 6.6, *argG* 1.6			
* dga* genes		*dgaB* ‐ 5.54, *dgaC* ‐ 5.73, *dgaD* ‐ 5.52		
* prd* genes		*prdA* 2.77, *prdB* 2.26, *prdD* 4, *prdE* 3.44, *prdF* 3.54	*prdB* 2.56, *prdD* 4.42, *prdE* 4.02	
* pot* genes	*potB* 2.08, *potD* 2.2			

## APPENDIX C

**Table C1:** Main characteristics of qPCR and PCR targeting specific genes.

Genes	Name	Primer reverse	Primer forward	Probe	Tm	References
16S rRNA gene (1500 bp)	16S	5′ TACGGTTACCT TGTTACGAC 3′	5′ GAGTTTGATC MTGGCTCAG 3′	NR	56	Minutillo et al. (2023)
		5′	5′	5′ FAM‐		
16S rRNA gene (157 bp)	16S rRNA gene of *C. difficile*	CCATCCTGTAC TGGCTCACCT 3′	TTGAGCGATT TACTTCGGTA AAGA 3′	CGGCGGACG GGTGAGTAA CG‐TAMRA 3′	55	Mutters et al. (2009)
				5′‐Hex‐		
*gluD* (116 bp)	Glutamate dehydrogena se	5′‐ CCTCTATAACT CTCATAGGTTC ‐3′	5′‐ AAAAGATGTA AATGTCTTCG AG‐3′	TTCATAAACT GCTGGTTCC ATACCT‐ BHQ2‐3	57	Adapted from Kouhsari et al. (2019)
		5′‐	5′‐	5′‐FAM‐		
*tcdA* (100 bp)	Toxin A	TTTACTAGATA AATCGCTCATA ATAG‐3′	ATATGAAGTA AGAATTAATA GTGAGG‐3′	AAGAACTTC TGGCTCACTC AGGTAA‐ TAMRA‐3′	57	
		5′‐	5′‐	5′‐CY5‐		
*tcdB* (110 bp)	Toxin B	TTTATAATACC CTTACTATTAA ATGC‐3′	GCTTCTAAGT CAGATAAATC AG‐3′	ACTTCTAGTG GTGATGCCT CCATAT‐ BHQ3‐3′	57	
		5′‐	5′‐			
*rnfG (164 bp)*	Rnf complex	TGTTACCAATG TCAACACCT‐3′	AGACCTTGGA GATGGTCTTA‐ 3′	NR	54	This study
*flaA (192 bp)*	electron transfer flavoprotein A	5′‐ ACTCCATAGTC AGCGATTTC‐3′	5′‐ TGTTGATGCT GGATGGATAG ‐3′	NR	55	This study
*eutA (154 bp)*	Ethanolamin e A	5′‐ AAGCCTTCCAC CTATATCCA‐3′	5′‐ AATATCAGGA AAAGGGGCTG‐3′	NR	54	This study
*eutB (140 bp)*	Ethanolamin e B	5′‐ GGAGCACCAT TTCTTATTGC‐ 3′	5′‐ CCAGTTGATG ACTCTGTTGA‐ 3′	NR	57	This study

Abrreviations: NR, not realized; qPCR, quantitative polymerase chain reaction.
