# Supplementary material for: Influence of microbiota on the growth and gene expression of Clostridioides difficile in an in vitro coculture model
Source: Microbiologyopen. 2024 Oct 15;13(5):e70001. doi: 10.1002/mbo3.70001 (PMC11633334; doi:10.1002/mbo3.70001)
Supplement: Supplementary file 1 — Supporting information. [file MBO3-13-e70001-s001.docx]

Supplemental Tables S1, S2, S3 and S4

Results from transcriptomic analysis of Clostridioides difficile in a coculture with faeces.

**Table S1 –**The results of the Deseq2 analysis are shown. The results are represented in log_2_fold change. There are four folders: analysis of 12h from analysis 1 and 2 (n=6 samples), analysis of 12h from analysis 2 (n=3), analysis of 24h from analysis 2 (n=3), analysis between 12h and 24h (n=3).

**Table S2 –**The significant results of the Deseq2 analysis are shown. The results are represented in log_2_fold change. There are four folders: analysis of 12h from analysis 1 and 2 (n=6 samples), analysis of 12h from analysis 2 (n=3), analysis of 24h from analysis 2 (n=3), analysis between 12h and 24h (n=3).

**Table S3** **–**Main genes of categories represented in log_2_fold change of all genes and of significant genes in different comparisons. Fourteen categories are represented: Sporulation associated genes, division cell associated genes, cwp associated genes, peptidoglycans associated genes, germination associated genes, virulence genes, motility genes, ethanolamine metabolism genes, ornithine metabolism genes, RNF system genes, iron metabolism, flavoprotein associated genes, biofilm associated genes, amino acids metabolism genes. Nine comparisons are shown: (1) Time impact: WOF 12h vs WOF 24h; F04 12h vs F04 24h; F03 12h vs F03 24h. (2) Microbial impact: F03 12h vs WOF 12h; F04 12h vs WOF 12h; F04 12h vs F03 12h and F03 24h vs WOF 24h; F04 24h vs WOF 24h; F04 24h vs F03 24h.

**Table S4** **–**Main significant modules and significant KO number represented in log2fold change of nine comparisons. Nine comparisons are shown: (1) Time impact: WOF 12h vs WOF 24h; F04 12h vs F04 24h; F03 12h vs F03 24h. (2) Microbial impact: F03 12h vs WOF 12h; F04 12h vs WOF 12h; F04 12h vs F03 12h and F03 24h vs WOF 24h; F04 24h vs WOF 24h; F04 24h vs F03 24h.
